# Prevalence and determinant factors of exclusive breastfeeding practices among mothers in Enderta woreda, Tigray, North Ethiopia: a cross-sectional study

**DOI:** 10.1186/s13006-014-0028-z

**Published:** 2015-01-20

**Authors:** Bahre Teka, Huruy Assefa, Kiday Haileslassie

**Affiliations:** Tigray Health Bureau, P.O.Box, 07, Mekelle, Tigray Ethiopia; Biostatistics and Epidemiology Unit, Department of Public Health, College of Health Sciences, Mekelle University, P.O.Box, 1871, Mekelle, Ethiopia; Reproductive and Human Nutrition Unit, Department of Public Health, College of Health Sciences, Mekelle University, P.O.Box, 1871, Mekelle, Ethiopia

**Keywords:** Exclusive breastfeeding, Tigray, Ethiopia

## Abstract

**Background:**

In Ethiopia national breastfeeding practice is poor because of traditional and cultural beliefs, low educational levels, heavy workload of mothers, poor sanitary conditions, type of assistance at delivery, duration of stay at home, ethnicity, poor maternal knowledge, age, parity, antenatal service utilization and place of delivery. This study is aimed to assess the prevalence and determinants of exclusive breastfeeding practice in mothers in Enderta woreda (district), Ethiopia.

**Methods:**

A community based cross-sectional study with multistage sampling method was used to select 541 mothers with children less than 24 months of age in Enderta woreda. Data was collected by administered structured questionnaire. Bivariate and Multivariable logistic regression was used to check the associations and controlling confounding variables.

**Result:**

A total of 530 mothers were included with a response rate 98%. The mean (± SD) age of the mothers was 26.9 *(±* 5.98*)* years*.* Majority of the mothers (70.2%) were practiced exclusive breastfeeding. Age of the mother (AOR 0.12; 95% CI: 0.02, 0.97), age of the child (AOR 0.52 95% CI: 0.27, 0.99) and postnatal care (AOR; 2.68; 95% CI: 1.44, 4.98) were found statistically significant with exclusive breastfeeding.

**Conclusion:**

The prevalence rate of exclusive breastfeeding was high in Enderta woreda. The age of the mother, age of the child and receiving postnatal care were the determinant factors for exclusive breastfeeding in the study area.

## Background

Only 39% of all infants are exclusively breastfed worldwide and the prevalence of exclusive breastfeeding rarely exceeds 30% in most regions in the developing world [1]. Globally, more than 10 million children under the age of five year die each year, 41% of these deaths occur in sub-Saharan Africa and 34% in south Asia. A major cause of death is inadequate breastfeeding practice in combination with high levels of disease [2].

In Ethiopia, as in other developing countries, diarrhea is a major contributor of morbidity and mortality in young infants and children, especially in urban areas, due to inappropriate breastfeeding practices [3]. About 58% of the child deaths are attributable to malnutrition, making this, the greatest single cause of child mortality and about 70% of infants are sub-optimally breastfed, another major contributor to infant mortality rate. Currently 24% of infant death is due to poor breastfeeding practices [4].

In Ethiopia national breastfeeding practices is poor because of traditional and cultural beliefs, low educational levels, heavy workload of mothers, poor sanitary conditions, type of assistance at delivery, duration of stay at home, ethnicity, poor maternal knowledge, age, parity, antenatal service utilization and place of delivery [5,6].

According to 2011 Ethiopian Demographic and Health Survey (EDHS), 52% of Ethiopian infants were exclusively breastfeeding at age of six months. The percentage of newborns who received prelacteal feeding in Ethiopia and Tigray were 27% and 25.6%, respectively. About 52% and 45% were put to breast within one hour in Ethiopia and Tigray, respectively [7].

The Ministry of Health of Ethiopia has tried to enhance optimal breastfeeding practice by developing training manuals and implementation guidelines on breastfeeding. These have been incorporated into the primary health care system in line with the health extension program but breastfeeding practice remains far from the global recommendation. The 2011 EDHS showed the proportion of infants less six months who received exclusive breastfeeding (EBF) as 52% [7] which improved slightly compared to 2005 EDHS (49%) [8]. Despite a few local studies conducted in Ethiopia, no sufficient study tried to identify the determinants of optimal breastfeeding practice in the study area. Therefore, this study is aimed to assess the prevalence and determinants of exclusive breastfeeding practice among mothers in Enderta Woreda (district), Tigray, north Ethiopia.

## Methods

### Study setting and participants

A community based cross-sectional study was conducted from February 2013 to April 2013 among mothers having children aged less than 24 months residing in Enderta Woreda. Enderta woreda is one of the 5 woredas of South East Zones of Tigray, which has a total population of 106,749 and from this population; 54,432 are females and 52,298 are males. In this woreda 15,583 children under 5 years and 25,065 women ages 15–49 years are living; and the expected number of pregnancies in a year is 3,629 with an expected delivery rate of 3,309.

To select study participants, a stratified sampling technique was used. Five kebeles (neighborhoods) were selected from 17 kebeles in the woreda using a simple random sampling technique. In the five selected kebeles a total of 1092 (203, 255, 190, 246 and 198 in each kebele) eligible mothers were available and a total of 541 (101, 126, 94, 122 and 98) eligible mothers from each respective kebeles were selected using population proportion to size (PPS). Each individual was selected using systematic sampling technique from the list of households. Households with children 0 to 24 months were identified by immunization cards from health facilities as well as house to house request (a list was obtained from Health Extension Workers) and eligible mothers were recorded and numbered. Mothers who had their children living with them were eligible and were interviewed using systematic random sampling technique until the predetermined sample size was obtained. The sample size was determined by using a single population proportion formula considering the following assumptions: Proportion of EBF 31%, 95% level of confidence and 5% marginal error. The final sample size was adjusted using the design effect of 1.5% and 10% non-response rate. Thus, the sample size required was 541 participants.

### Measurements

Data on socio-demographic information, obstetric factors like parity, child and breastfeeding practice factors, maternal knowledge about breastfeeding were collected using a pre-tested and structured questionnaire. Exclusive breastfeeding is defined as only breast milk was given in the 24 hours preceding the interview. Since we included children less than 24 months, the prevalence of exclusive breastfeeding was determined by using birth dietary recall method. The mothers were asked if any liquid or food item had been given to the children. This also allows avoiding overestimation in determining prevalence of EBF. The prevalence of exclusive breastfeeding was calculated as the ratio of children below 6 months who fed only on breast milk in the 24-hours to the total number of children in the same age group. To calculate the median durations of exclusive breastfeeding practice, mothers were also asked to answer “for how many months did they feed their child with breast milk only?”

Prelacteal feeding is feeding of an infant with something other than breast milk during the first three days of life. Colostrum is the first thick yellow milk secreted by the breast in the last few weeks’ pregnancy and the first two to three days after child birth, until lactation is established. Timely initiation of breastfeeding is defined as the neonate on the mother’s breast to suckle within one hour (including one hour). Sufficient knowledge on benefit of breastfeeding defined as “When the respondents identified correctly at least five out of ten statements about the benefits of exclusive breastfeeding to child and mother”.

From each selected household one eligible child age less than 24 months and having biological mother at the time of survey was selected. The youngest child was selected from mothers who have two children less than 24 months in one household. For selected households not having an eligible child, the next household was selected.

### Data analysis

Data were entered into Epi Info version 3.5.1 and exported to SPSS version 20.0 statistical package for analysis. The results were presented in the form of tables and text using frequencies and summary statistics such as mean and standard deviation for continuous variables and percentages for categorical variables to describe the study population in relation to relevant variables. The data were analyzed using logistic regression to determine the determinant factors on the outcome variable and to control confounding. All variables having a p-value ≤ 0.05 in the bivariate analysis were further entered into multivariable logistic regression model. Variables having p-value ≤ 0.05 in the multivariable logistic regression were statistically significant. Crude and adjusted odds ratios with their 95% confidence intervals were calculated. The Hosmer and Lemeshow goodness of fit test was used to assess whether the necessary assumptions for the application of multiple logistic regression were fulfilled and p-value ≥ 0.05 was considered a good fit.

### Ethical considerations

Ethical clearance was obtained from Ethical Review Committee of Mekelle University, College of Health Sciences and Department of Public Health. An official permission letter was obtained from Enderta Woreda Health Office. Informed verbal consent was obtained from study participants in their local language after explaining the purpose of the study, potential risks and benefits of partaking in the study, and the right to withdraw from the study at any time. The participants were also assured that the data was confidential.

### Consent

Written informed consent was obtained from the patient’s guardian/parent/next of kin for the publication of this report and any accompanying images.

## Results

### Sociodemographic characteristics of the participants

From the total of 541 eligible mothers, 530 mothers were included in the analysis with a response rate 98%. The mean (± SD) age of the mothers was 26.9 *(±*5.98) years*.* The mean (± SD) age of the children was 9.6 (±5.88) months. More than half of the children (55.3%) and women (55.3%) were females and illiterate, respectively. Majority of the mothers were married (87.7%), Orthodox in religion (97.5%) and a house wife (87%). The majority of house wives exclusively breastfed 334 (72.5%). When married 333 (71.6%) with a 200-700birr household income, 262 (69.5%) mothers were practicing EBF. The median household monthly income was 600 birr (IQR = 350 birr) (Table 1).

**Table 1:** **Socio-demographic characteristics of exclusive breastfeeding mothers (n = 530) in Enderta woreda, Tigray, North Ethiopia 2013**

**Variable**	**N (%)**	**EBF (%)**
**Age of mother (years)**		
15-19	47 (8.9)	36(76.6)
20-24	144 (27.2)	103(71.5)
25-29	160 (30.2)	114(71.2)
30-34	106 (20)	75(70.8)
35-39	55 (10.4)	35(63.6)
>=40	18 (3.4)	9(50)
**Age of child in month**		
=<6	200 (37.7)	161(80.5)
7-12	193 (36.4)	123(63.7)
13-18	73 (13.9)	46(63)
19-24	64 (12.1)	42(65.6)
**Sex of child**		
Male	218 (41.1)	158(72.5)
Female	312 (58.9)	214(68.6)
**Marital status**		
Married	465 (87.7)	333(71.6)
Single	2 (0.4)	2(100)
Divorced	42 (7.9)	23(54.8)
Widowed	12 (2.3)	5(41.7)
Separate	9 (1.7)	9(100)
**Educational status (mother)**		
Illiterate	293 (55.3)	192(65.5)
Read and write	59 (11.1)	45(76.3)
Attend formal school	178 (33.6)	135(75.8)
**Occupation (mother)**		
Housewife	461 (87)	334(72.5)
Farmer	40 (7.6)	22(55)
Daily laborer	29 (5.5)	16(55.2)
**Household monthly income**		
200-700	377 (71.1)	262(69.5)
701-1200	123 (23.2)	85(69)
1201-1700	12 (2.3)	9(75)
>=1701	18 (3.4)	16(88.9)

### Obstetric and health service related characteristics of the participants

From the 459 (86.65%) mothers visited health institution for ANC, 354 (66.8%) of the mothers were visited the health institution for ANC at least once. Majority 429 (93.5%) of the mothers were counseled on breastfeeding during ANC visit. Three hundred forty (64.2%) of the mothers were delivered at home and more than half 277 (52.3%) of the mothers were visited health institution for postnatal care (Table 2).

**Table 2:** **Distribution of mothers by obstetric history and health service related (n = 530) in Enderta woreda, Tigray, north Ethiopia 2013**

**Variable**	**N (%)**	**EBF (%)**
**Parity**		
1-2	179 (33.8)	128 (71.5)
3-4	211 (39.8)	148 (70.1)
5-6	104 (19.6)	70 (67.3)
>=7	36 (6.8)	26 (72.2)
**ANC visit for last child**		
Yes	459 (86.6)	325 (71)
No	71 (13.4)	47 (66)
**Number of ANC visits for last child**		
Zero	71 (13.3)	47 (66)
One	354 (66.8)	240 (68)
Two to three	95 (17.9)	78 (82)
Four and above	10 (1.9)	7 (70)
**Health education/counseling on breastfeeding during ANC**		
Yes	429 (93.5)	302 (70)
No	30 (6.5)	23 (77)
**Place of delivery**		
Home	340 (64.2)	220 (65)
Hospital	46 (8.7)	39 (85)
Health center	144 (27.1)	113 (78)
**Postnatal care**		
Yes	277(52.3)	205(74)
No	253(47.7)	167(66)

Of the mothers visited health institution for ANC, 325 (71%) were practicing EBF and from the total mothers visited health institution for ANC at least once, 240 (68%) were practicing EBF. Of the mothers counseled on breastfeeding during ANC, 302 (70%) of the mothers were practicing EBF and from the mothers delivered at home, 220 (65%) were practicing EBF. And from the mothers attended postnatal care, 205 (74%) were practicing EBF (Table 2).

### Breastfeeding practices

Greater number 362(68.3%) of the mothers were initiate breastfeeding immediately. Majority 491(92.6%) of the mothers had fed colostrums and only 68(12.8%) of the mothers gave prelacteal feed. Three hundred ninety seven (74.9%) of the mothers were breastfeeding less than 10 times per day (Table 3).

**Table 3:** **Distribution of mothers by their exclusive breastfeeding experiences (n = 530) in Enderta woreda, Tigray, north Ethiopia, 2013**

**Variable**	**N (%)**	**EBF (%)**
**Initiation of breastfeeding**		
Immediately	362 (68.3)	264 (73)
Within one hour	127 (24)	83 (65)
1-24 hour	40 (7.5)	25 (63)
After 24 hour	1 (0.2)	
**Colostrum discarded**		
Yes	491 (92.6)	350 (71)
No	39 (7.4)	22 (56)
**Prelacteal feeding**		
Yes	68 (12.8)	
No	462 (87.2)	372 (81)
**Frequency of breastfeeding per day**		
<10	397 (74.9)	274 (69)
>=10	133 (25.1)	98 (74)
**Utensil used to feed the child**		
Bottle feed	1 (0.3)	1 (100)
Cup and spoon	226 (67.7)	145 (64.2)
Hand	107 (32)	54 (50.5)
**Age of young child**		
< 6 months	200 (37.7)	161 (81)
>= 6 months	330 (62.3)	211 (64)
**knowledge about the benefits of breastfeeding**		
Yes	335 (61.92)	258(77.01)
No	206 (38.08)	66(32.04)

Large number of mothers 372 (70.2%) were practiced exclusive breastfeeding. From the mothers initiated breastfeed immediately, 264(73%) were practicing EBF. From the mothers fed colostrums, 350(71%) were practicing EBF (Table 3).

### Determinants of exclusive breastfeeding

Variables which were statistically significant at p-value ≤ 0.05 in the bivariate logistic regression included in the multivariable logistic regression. These variables were age of mother, religion, place of hand washing, youngest child age, number of ANC visits, place of delivery, postnatal care, opinion on the time to feed breast milk only, received advice to start and continue breastfeeding, opinion on advantages of breastfeeding to mother, breastfeeding prevents pregnancy, and knowledge on breastfeeding as a contraceptive (Table 4).

**Table 4:** **Multivariable logistic regression analysis of factors associated with exclusive breastfeeding among mothers (n = 530) with exclusive breastfeeding, Enderta woreda, Tigray, north Ethiopia, 2013**

**Variables**	**Yes (%)**	**Crude odds ratio (95% Confidence interval)**	**Adjusted odds ratio (95% Confidence interval)**	
**Age of mother**	15-19	36 (76.6)	1	1
	20-24	103 (71.5)	0.77 (0.36, 1.65)	0.38 (0.07-2.04)
	25-29	114 (71.2)	0.76 (0.36, 1.61)	0.31 (0.06, 1.68)
	30-34	75 (70.8)	0.74 (0.33, 1.64)	0.47 (0.09, 2.61)
	35-39	35 (63.6)	0.54 (0.22, 1.27)	0.28 (0.05, 1.72)
	>=40	9 (50.0)	0.31 (0.09, 0.96)	0.12 (0.02, 0.97)*
**Religion**	Orthodox	367 (71)	1	
	Muslim	5 (38.5)	0.26 (0.08, 0.79)	
**Place of hand wash**	not washing	45(49.5)	1	
	in the house	162 (73.3)	2.81 (1.69, 4.66)	
**Child age**	=<6	161 (80.5)	1	1
	7-12	123 (63.7)	0.43 (0.27, 0.67)	0.52 (0.27, 0.99)*
	13-18	46 (63.0)	0.40 (0.23, 0.75)	0.62 (0.25, 1.58)
	19-24	42 (65.6)	0.46 (0.25, 0.86)	0.86 (0.30, 2.44)
**Number of ANC visit**	1	240 (67.8)	1	
	2-3	85 (81)	2.02 (1.18, 3.45)	
**Place of delivery**	home	220 (64.7)	1	
	hospital	39 (84.8)	3.04 (1.32, 7.00)	
	health center	113 (78.5)	1.99 (1.26, 3.14)	
**Postnatal care**	No	205 (74)	1	1
	Yes	167 (66)	0.68 (0.47, 0. 99)	2.68 (1.44, 4.98)*
**Opinion on the time to feed breast milk only**	6 month	215 (74.7)	1	
	1-2 year and 6 month	157 (64.9)	0.63 (0.43, 0.91)	
**Person advised to start and continue breast feed**	health worker	199 (76)	1	
	husband	173 (64.6)	0.58 (0.39, 0.84)	
**Opinion on advantage of breastfeeding to mother**	grows best	91 (73.4)	1	
	prevent disease	131 (66.8)	0.63 (0.42, 0.95)	
	provide complete nutrition	95 (72)	0.79 (0.44, 1.42)	
	Don’t know	40 (64.5)	0.97 (0.05, 2.46)	
**Breastfeeding prevents pregnancy**	No	83 (63.4)	1	
	Yes	289 (72.4)	1.52 (1.00, 2.31)	
**Knowledge on criteria breastfeed used as contraceptive**	If fully breastfeed	82 (78.8)	1	
	BF used as contraceptive	46 (71.9)	0.69 (0.33, 1.41)	
	Using breastfeeding	32 (64)	0.48 (0.23, 1.01)	
	I don’t know	212 (67)	0.57 (0.34, 0.964)	

Note: *: statistically significant.

Significant variables at p-value ≤ 0.05 were taken to the multivariate logistic regression analysis in order to develop the full model for the study. In the multivariate logistic regression analysis only age of the mother, age of the youngest child and postnatal care were found statistically significant with exclusive breastfeeding.

Controlling for the age of youngest child and maternal age, mothers received postnatal care were 2.68 (AOR 2.68 95% CI: 1.44, 4.98) times more likely to breastfed their child compared to those who did not receive postnatal care (Table 4).

After controlling for the other variables maternal age and postnatal care, mothers with 7–12 month age children were 48.4% (AOR 0.516; 95% CI:0.267, 0.995) less likely to exclusively breastfeed their child compared to those whose child was less than or equal to six months.

Controlling for the other variables, mothers aged forty years or over were 87.9% (AOR: 0.121; 95% CI 0.015, 0.970) less likely to exclusively breastfed their child than mothers aged 15 to 19 years old (Table 4).

## Discussion

This study revealed that, the prevalence rate of exclusive breastfeeding was 70.02%. This finding was less than the study done by Essential Service for Health in Ethiopia (ESHE) in Amhara (87%) and Oromiya (79%) [9]. This might be due to the fact that in Tigray region, there are less non-governmental organization employed to work with local health offices on EBF as one strategy to reduce child mortality in comparable to Amhara and Oromiya regions. However the finding was higher than the EDHS 2011(52%) [7], the study conducted in Ghana (51.6%) [10] and the study done in Thailand 54% [11]. This might be due to the fact that now in Ethiopia the new health extension package strategy is effective and, another possible reason might be the studies were done some years before the many improvements implemented in Ethiopia by the Ministry of Health in the area of child and maternal health.

In this study mothers were assessed whether they have adequate knowledge about the benefits of breastfeeding or not. Benefits of breastfeeding such as it is used as contraceptive effect, nutritious importance, disease protection, and mother to child relationship bonding and other related advantages were also mentioned in different ways to ascertain the level of mothers’ knowledge. The study was revealed that, almost 77% have sufficient knowledge on the benefits of breastfeeding for its prevention for pregnancy. This finding was found to be higher as compared to study done in Jimma (67.2%) [12]. This could be due to the fact that the birth rate in Jimma is higher than the study area.

This study revealed that age of the child was a determinant of exclusive breastfeeding. Mothers with a child aged 7–12 months were 48.4% less likely to exclusively breastfed their child compared to those mothers who have child in the age group of less than or equal to six months. This finding was in agreement with a study conducted in Ethiopia [13]. This could be probably explained by the short birth interval/spacing and other economic factors. It can also be attributed to the fact that postpartum care traditionally is given in the first few months when mothers are confined at home, creating an opportunity to exclusively breastfeed their child.

This study was also consistent with a study done in Guatemala [14] which showed that child’s age was statistically associated with exclusive breastfeeding. This similarity might be observed due to the similarity between Ethiopia and Guatemala. They have similar socio-economic, socio-demographic, child health and maternal health related factors.

A study on exclusive breastfeeding done in Mekelle [15] revealed that delivery place was a determinant factor for exclusive breastfeeding, however this study showed that delivery place was not statistically associated with exclusive breastfeeding. The difference might be because the government of Ethiopia has made an impact on maternal health by implementing the health extension package program, and a campaign to make the maternal health service 100% nationally available by having a slogan “Mother should not die while giving birth”.

Postnatal care is an independent determinant factor for exclusive breastfeeding; showing that mothers received postnatal care were more likely to exclusively breastfeed their child. Similar studies showed that in Africa two-third of women do not receive post-natal care and according to EDHS 2011 in Ethiopia 90% of women do not receive postnatal care [7]. The low uptake could impact excusive breastfeeding rates as.

This study also showed that number of ANC visits, place of delivery and maternal education were significantly associated with exclusive breastfeeding on the bivariate analysis but not on the full model (the multivariable logistic regression). But a study done by Yirga [16] showed that the number of ANC visit, place of delivery and maternal education were determinant factors for exclusive breastfeeding. This difference might be the result in socio-demographic characteristics of the study participants and number of health facility infrastructure difference of the study areas. The study in Addis Ababa was done some years before when the government paid little attention to maternal health in the study area.

Since this study is a cross-sectional descriptive approach, it has a limitation to formulate a casual association as to how and when the associations are established. Recall, since birth for exclusive breastfeeding, the time of initiation, the duration and patterns of breastfeeding are subject to potential recall bias. There is also a limitation using logistic regression for cross-sectional study. The prevalence ratio can be overestimated by odds ratio and controlling for confounding is not equivalent for the two measures [17].

## Conclusion

Prevalence of exclusive breastfeeding practice in Enderta woreda was lower than the WHO recommendations Postnatal care, maternal age and youngest child age were determinant factors for exclusive breastfeeding. Maternal knowledge on the benefits of breastfeeding for the unwanted pregnancy was high. Moreover their knowledge is dependent on the state of educational status and attendance of antenatal and postnatal care.

## Abbreviations

ANC: Antenatal care
AOR: Adjusted odds ratio
CI: Confidence interval
COR: Crude odds ratio
EBF: Exclusive breastfeeding
EDHS: Ethiopian Demographic and Health Survey
IQR: Inter quartile range
PPS: Population proportion to size
SD: Standard deviation
SPSS: Statistical Package for Social Science
WHO: World Health Organization
